# Supplementary material for: Optically Controlled Dissolution Kinetics of Vaterite Microcapsules: Toward Novel Crystal Growth Strategies
Source: Cryst Growth Des. 2023 Sep 26;23(11):8009–17. doi: 10.1021/acs.cgd.3c00799 (PMC10626575; doi:10.1021/acs.cgd.3c00799)
Supplement: Supplementary file 1 — cg3c00799_si_001.pdf [file cg3c00799_si_001.pdf]

Optically Controlled Dissolution Kinetics of Vaterite Microcapsules: Towards Novel Crystal Growth Strategies

Andrei Ushkov, Andrey Machnev, Pavel Ginzburg*

School of Electrical Engineering, Tel Aviv University, Tel Aviv 69978, Israel

KEYWORDS: drug carriers, vaterite, optical tweezer, micropipette, particle-fluid interactions, 3D micromanipulation.

Supplementary material

Diffusion-limited dissolution model

The steady-state ions concentration around the vaterite follows the law [1][2]:

$$C(r) = \begin{cases} C_s, & 0 \leqslant r \leqslant R \\ \dfrac{R(C_s - C_{bulk})}{r} + C_{bulk}, & r \geqslant R \end{cases} \quad \text{(S1)}$$

where C(r) is a species concentration at a distance r from the particle center, R is the particle radius, C_s is a vaterite solubility, and C_{bulk} is a species concentration far away from the particle. As the concentration at the vaterite/liquid interface is equal to the vaterite solubility, the particle dissolution process is limited by the transport of dissolved species away from the interface via diffusion, and is

therefore called a diffusion-limited process. Applying Fick's first law for C(r) in Eq.(S1) and the integration of flows of matter around the sphere yields the particle size-dependent dissolution rate [2]:

$$\frac{dr}{dt} = \frac{DV_m\left[C_{bulk} - C_s\right]}{r} \cdot \ln(2) \equiv \frac{B}{r} \qquad \text{(S2)}$$

where D is an effective electrolyte ions diffusion constant, V_m is a vaterite molar volume, and ln(2) takes into account the proximity of the particle to the substrate [3].

Calcium carbonate molar volume V_m=37.71*10^{-6} m^3/M [4].
Ca^{2+} diffusion coefficient D_{Ca}=0.79*10^{-9} m^2/s [5].
CO_3^{2-} diffusion coefficient $D_{CO3}=D^0_{CO3}(\frac{T}{T_{CO3}}-1)^{\gamma}$ = 0.8012*10^{-9} m^2/s, where D^0_{CO3} = 5.4468*10^{-9} m^2/s, T_{CO3}=210.2646 K, T=298 K, γ=2.1929 [6].
Effective diffusion coefficient of a symmetric electrolyte $CaCO_3$: D=2 $D_{Ca}D_{CO3}/(D_{Ca}+D_{CO3})$=0.796*$10^{-9}$ m^2/s [7].

Solubility of vaterite C_s without taking into account a complex atmosphere/DI water/$CaCO_3$ chemical equilibria: $C_s=\sqrt{K_v}$=0.11 M/m^3, where log(K_v)=-172.1295-0.077993*T+3074.688/T+71.595*log(T), T=298 K [8].

The concentration of diffused species in the bulk of DI water C_{bulk} was estimated as zero, taking into account a big volume of Petry dish (~16 ml).

Therefore, C_{bulk} - C_s= -0.11 M/m^3.

Diffusion-controlled dissolution model: Eq.(S2).

Thus, the coefficient B, calculated via the data above, is B_0=-0.0023 m^2/s.
The coefficient B, obtained via the fitting of experimental data (Fig.7c of the main text), is B_{exp}=-0.0019 m^2/s.

References

[1] Kralj D, Brečević L, Nielsen AE. Vaterite growth and dissolution in aqueous solution II. Kinetics of dissolution. Journal of Crystal Growth. 1994. pp. 269–276. doi:10.1016/0022-0248(94)90067-1
[2] Nielsen AE. Transport control in crystal growth from solution. Croatica chemica acta. 1980;53: 255–279.
[3] Bobbert, P. A., M. M. Wind, and J. Vlieger. "Diffusion to a slowly growing truncated sphere on a substrate." *Physica A: Statistical Mechanics and its Applications* 141.1 (1987): 58-72.
[4] Wyckoff, R.W.G. *Crystal Structures*, Vol. 2. 2nd Edition, John Wiley & Sons, Inc., New York, London, Sydney, 1964.
[5] Cussler, Edward Lansing. *Diffusion: mass transfer in fluid systems*. Cambridge university press, 2009.
[6] Zeebe, Richard E. "On the molecular diffusion coefficients of dissolved CO2, HCO3-, and CO32- and their dependence on isotopic mass." *Geochimica et Cosmochimica Acta* 75.9 (2011): 2483-2498.

[7] Nielsen, A. E. "Transport control in crystal growth from solution." *Croatica Chemica Acta* 53.2 (1980): 255-279.
[8] Plummer, L. Niel, and Eurybiades Busenberg. "The solubilities of calcite, aragonite and vaterite in CO2-H2O solutions between 0 and 90 C, and an evaluation of the aqueous model for the system CaCO3-CO2-H2O." *Geochimica et cosmochimica acta* 46.6 (1982): 1011-1040.
